# Supplementary material for: Are carbon emissions trading and green financial instruments synergistic? -Comprehensive quantitative research based on content analysis
Source: PLoS One. 2024 Mar 7;19(3):e0298601. doi: 10.1371/journal.pone.0298601 (PMC10919601; doi:10.1371/journal.pone.0298601)
Supplement: S1 Data — (ZIP) [file pone.0298601.s001.zip › Data/地级市Z_F工作报告.zip!/║╙─╧/╨φ▓2╩╨/└┤╫╘╣1⁄2╓┌║┼í╛┬φ┐╦╩2╛▌═°í┐_2012.docx]

各位代表：

现在，我代表市人民政府，向大会作工作报告，请予审议，并请各位政协委员和其他列席会议人员提出意见。

一、过去五年工作回顾

2007年以来，在省委、省政府和市委的正确领导下，市政府团结带领全市人民，锐意进取，开拓创新，真抓实干，圆满完成了市五届人大确定的各项目标任务，改革开放和各项建设取得了新的重大成就。2011年，全市生产总值完成1588.7亿元，是2006年的1.9倍；财政一般预算收入74.2亿元，是2006年的2.9倍；全市粮食总产277.2万吨，实现“八连增”；城镇累计新增就业41.9万人，农村劳动力累计转移就业139.7万人，动态消除了零就业家庭；城镇居民人均可支配收入17503元，农民人均纯收入8650.7元，分别是2006年的1.9倍和2倍。我们科学谋划制定了“十二五”发展规划，进一步明确了在中原经济区建设中的功能定位，初步探索了以新型城镇化为引领的“三化”协调发展之路，巩固和扩大了应对国际金融危机冲击成果，持续了经济社会好的发展趋势、态势和气势，实现了“十二五”良好开局。

五年来，我们主要做了以下工作：

（一）加强运行调节，促进经济平稳较快发展。面对严峻复杂的经济形势，我们结合实际，科学决策，沉着应对国际金融危机冲击，牢牢把握经济工作主动权。持续实施投资促进计划，增强经济增长拉动力。2011年，全社会固定资产投资完成938.7亿元，是2006年的3.2倍，许继风电产业园、许昌卷烟厂易地技术改造等一大批重点项目相继开工建设。大力实施重点企业服务计划，稳定经济增长基础。建立完善政银企洽谈会和政策咨询会制度，有针对性地开展企业服务，2011年，规模以上工业增加值894.7亿元，是2006年的2.9倍。扩大居民消费需求，增强经济增长内生动力。2011年，社会消费品零售总额414.9亿元，是2006年的2.5倍。这些措施扭转了经济增速下滑趋势，加强了薄弱环节，既战胜了特殊困难、有力地保障和改善了民生，又为长远发展奠定了坚实基础。

（二）坚持新型城镇化引领，统筹城乡发展。着力推进中心市区、县（市）城区、中心镇区和新型农村社区协调发展，城镇化率达到43%。坚持实施中心带动，五年累计实施城建重点项目和城中村改造项目409个，完成投资270.5亿元，开工建设安置房303.2万平方米。全面启动许昌新区建设，拓展了城市发展空间；许都大剧院、博物馆、体育馆等一批标志性工程建成投用，市区八一路、文峰路、新兴路等主干道完成改造提升，城市功能日益完善；许昌至长葛、鄢陵、襄城快速通道建成投用，城市组团联系更加紧密；初步形成了以零距离换乘中心为标志的综合交通体系，交通区位优势更加凸显。坚持城乡统筹，确定实施了30个中心镇区和一批新型农村社区试点建设。不断完善农村基础设施，累计解决农村安全饮水97.4万人，新改建农村公路3251公里，建设户用沼气28.3万口，完成农村改厕61.1万户。南水北调中线及配套工程进展顺利，丹江口库区移民16514人安置任务圆满完成。切实加强城市管理，持续提升城市“三化”管理水平，深入开展全国文明城市创建活动，先后荣获“国家森林城市”、“国家卫生城市”、“全国绿化模范城市”、“中国花木之都”、“全国文明城市创建工作先进城市”、“中国金融生态城市”、“河南省数字化城市管理示范城市”。

（三）推进经济结构调整，加快转变发展方式。坚持保增长与调结构相结合，着力解决制约经济发展的结构性矛盾。全市二、三产业增加值占GDP的比重达到89%，比2006年提高4个百分点；税收占财政一般预算收入的比重达到78.9%，比2006年提高3.4个百分点。进一步完善现代工业体系，谋划建设了“一带十区二十个产业集群”，中原电气谷上升为省级战略，经济开发区晋升为国家级经济技术开发区。培育壮大了三大主导、四大特色和五大战略性新兴产业，工业增加值达到1044.7亿元，是2006年的2.1倍，4家企业主营业务收入超过100亿元，上市公司达到7家，创河南省省长质量奖1个，河南省名牌产品43个；大力发展现代农业，加快农业结构调整，花木、蔬菜、中药材等特色农业持续发展，畜牧业产值占农业产值的比重达到48%，市级以上农业龙头企业发展到245家，其中省级以上41家，总数居全省第2位；加快发展物流、文化旅游、金融等现代服务业，许昌银行、市农村信用社等地方金融机构规模迅速发展壮大，交通、光大等4家外埠股份制银行入驻许昌；不断完善自主创新体系，提升科技创新能力，科技进步对经济增长的贡献率达到55%，连续10年荣获“全国科技进步先进市”。扎实推进节能减排，大力淘汰落后产能，积极推进环境综合整治，化学需氧量和二氧化硫排放量分别削减15.14%和20.59%。

（四）深化改革开放，增强经济社会发展活力。努力消除体制障碍，不断提高对外开放水平。累计完成企事业单位改革改制217家，煤炭企业兼并重组和国有、集体企业改革基本完成，成功推动了许继集团的战略重组，实现了企业跨越式发展；积极推进重点领域改革，教育、文化、卫生、财政、金融等领域改革取得重大进展，政府机构改革如期完成，农村改革不断深化，管理体制改革有序推进。提升开放带动能力，成功引进国家电网、中国平安等一批战略投资者，许昌出入境检验检疫局成功获批，累计引进市外资金2211亿元，新批准外商投资企业73家，实际利用外资9.7亿美元，成为国家级外贸转型升级专业型示范基地和河南省发制品出口基地；不断扩大出口规模，累计出口45.8亿美元，是上个五年的2.9倍。

（五）加快发展社会事业，切实保障和改善民生。突出发展和民生两大主题，坚持每年实施一批民生工程，持续加大民生投入。五年来，全市用于改善民生的财政支出累计达293亿元，年均增长35.9%。加快发展教育事业，许昌电气职业学院、许昌陶瓷职业学院申报成功，人民群众关心的入学难、大班额等问题得到有效缓解，累计完成中小学危房改造及校舍安全工程49.7万平方米，建成公办幼儿园41所，“两免一补”及义务教育经费保障政策受益学生达到536.3万人次，16万人次农村贫困寄宿生享受生活补助，城镇学校共接收3.7万多名进城务工农民子女接受义务教育；全面推进医疗卫生事业，初步建立了基本药物制度，有效防控了艾滋病、手足口病等重大疫情，全市新农合参合率达97.4%，建设改造了5所县医院、31所乡镇卫生院、11所社区卫生服务中心，建成了2300所行政村标准化卫生室；不断完善社会保障体系，提升养老、医疗、住房等保障水平，建设各类保障性住房33252套，全面建立孤儿生活保障制度，城乡低保实现应保尽保，城市低保标准提高到每人每月210元以上，农村低保标准提高到每人每年1200元以上，五保集中供养率达到60.8%；持续改善居民生产生活条件，打通了一批断头路，建成了一批游园广场。社会福利院、群众艺术馆、残疾人康复中心等一大批社会事业项目建成投用，实现了垃圾无害化处理和集中供热。完成了99个贫困村整村推进建设任务，解决了近10万人的脱贫问题。文化、人口、体育、广播电视等各项社会事业全面发展，人民群众安居乐业。

（六）加强政府建设，推进民主法制。坚持依法行政，自觉接受人大及其常委会的监督，支持政协参政议政，认真办复人大代表议案建议和政协委员提案1495件；深入学习实践科学发展观，坚持“四个重在”实践要领，扎实开展“强两基、重民生、转方式、促发展”主题活动；切实推进“五个坚持、两转两提”，大力倡导清新简约、务本责实的良好政风；加快行政审批制度改革，行政审批事项由2006年的268项精简至193项；行政监察和审计监督更加有力，政务公开不断扩大；“五五”普法任务圆满完成；社会治安防控网络日益完善，矛盾纠纷调处机制逐步健全，安全生产监管不断加强，连年荣获“全省平安建设工作先进省辖市”、“全省食品安全优秀城市”；军政军民、民族、宗教、外事、侨务、对台工作全面加强，荣获“全国民族团结进步模范集体”，连续3届荣获“全国双拥模范城市”；地方史志、机关事务、档案、统计、地震、气象等工作都取得了新的成绩。

各位代表，这五年，是许昌克难攻坚、砥砺奋进、成就显著的五年；这五年，是许昌昂首阔步、奋发有为、亮点纷呈的五年；这五年，是许昌发展最快、变化最大、惠民最多的五年。这些成就的取得，是省委、省政府和市委正确领导的结果，是市人大、市政协监督支持的结果，是全市广大干部群众群策群力、艰苦奋斗的结果。在此，我代表市人民政府，向全市广大干部群众、驻许部队、武警官兵、公安民警表示崇高敬意！向所有关心、支持许昌现代化建设的各界人士表示衷心感谢！

回顾过去五年，我们深深体会到，做好政府工作，必须坚持把贯彻科学发展观与立足许昌实际结合起来，求客观规律之真，务科学发展之实；必须坚持把注重持续与善于统筹结合起来，突出新型城镇化的引领作用，持续探索走好“两不三新、三化协调”科学发展路子；必须坚持把深化改革开放与创新发展结合起来，牢牢把握加快改革创新这一强大动力，以改革开放的新突破，推动经济社会发展新跨越；必须坚持把加快发展与改善民生结合起来，着力在加快发展中改善民生、在改善民生中凝聚合力。

我们也清醒地认识到，许昌发展还面临不少问题：经济结构不优、服务业比重偏低、要素约束趋紧，加快发展方式转变更加紧迫；社会事业发展还比较滞后，公共服务能力和社会管理水平亟待提升；产业集聚度不够，重大项目较少，开放水平不高，发展的后劲、活力有待增强；一些干部科学运作、破解瓶颈的能力有待提高。对此，我们将高度重视，统筹协调，认真解决。

二、今后五年的总体要求和目标任务

今后五年，是我市圆满完成“十二五”规划、全面建设小康社会的重要时期，也是推进各项事业跨越发展的关键时期。世界经济的大调整、大变革、大发展，为我们承接产业转移提供了更加有利的条件；建设中原经济区战略的深入实施，为我们发展带来了更加难得的机遇；许昌多年来的改革发展成果积淀，为今后蓄势崛起奠定了更加坚实的基础。我们要认清新形势，抢抓新机遇，发挥新优势，巩固当前经济社会发展的好形势，推动全市经济社会又好又快发展。

按照市六次党代会提出的指导思想，今后五年政府工作的总体要求是：深入贯彻落实科学发展观，坚持“四个重在”实践要领，以科学发展为主题，以加快经济发展方式转变为主线，以加快新型城镇化为战略重点，围绕“两高两好”总体目标，着力“三化”协调，着力项目带动，着力改革开放，着力创业创新，着力改善民生，致力走出一条不以牺牲农业和粮食、生态和环境为代价的新型城镇化、新型工业化、新型农业现代化“三化”协调科学发展的路子，奋力在中原经济区建设中开创率先崛起、富民兴许新局面。

今后五年的奋斗目标是：经济发展速度和城乡居民收入增速高于全省平均水平，发展质量和生态环境好于全省平均水平。到2016年，全市生产总值突破2400亿元，年均增长10%以上；人均生产总值突破5万元，处于全省领先位置；公共财政预算收入突破150亿元，年均增长16%以上；城镇化率年均提高2个百分点左右，达到52%以上；基本公共服务水平和均等化程度全面提高，城乡居民收入与经济发展同步增长，为到2020年建成更高水平的小康社会奠定更加坚实的基础。

实现上述目标，事关全市人民福祉，事关许昌经济社会发展全局。我们将按照市六次党代会的总体部署，牢记使命，求真务实，顽强拼搏，加倍努力，不负众望，致力在加快转变经济发展方式、构建现代城镇体系、完善现代产业体系、推进改革开放、促进文化大发展大繁荣、保障和改善民生等方面取得新的突破。

三、2012年政府主要工作

根据今后五年的目标和任务，2012年我市经济社会发展的主要预期目标是：生产总值增长11%；公共财政预算收入增长16%；全社会固定资产投资增长22%；社会消费品零售总额增长16%；规模以上工业增加值增长16%；外贸出口总额增长19%，实际利用外资增长20%；城镇居民人均可支配收入实际增长11%，农民人均纯收入实际增长11%；城镇新增就业6.2万人以上；人口自然增长率控制在6.3‰以内；居民消费价格涨幅4%左右；城镇化率提高2个百分点；节能减排完成省定目标。

按照市委经济工作会议部署，围绕上述目标，今年将重点抓好以下6个方面的工作：

（一）强化引领作用，大力推进新型城镇化

认真落实市六次党代会、全市城镇化工作会议精神，把加快推进新型城镇化作为扩内需、调结构、促转型、惠民生的综合性举措，加快构建现代城镇体系。

进一步完善城乡规划体系。树立全域规划理念，推进“五规合一”，促进城乡规划有机融合，扎实推进中心市区组团式、县市城区内涵式、中心镇区特色式、新型农村社区集聚式发展。抓紧城市总体规划修编，高标准编制城市专项规划、重要区域城市设计和控制性详细规划，提高市区“二级组团”规划建设水平。加强镇村规划编制和管理，突出抓好中心镇区和新型农村社区规划，力避骑路规划、沿路建设老模式。坚持规划两级审批和“三位一体”审批机制。完善规划监督机制，综合运用法律、经济、行政、技术等多种措施，做好已批规划的过程监管。建立完善城乡规划公示、企业诚信管理、联动协调处理等制度，加大规划执法力度，坚决有效制止私搭乱建，确保规划有效执行、落到实处。

全力推进新型农村社区建设。把新型农村社区建设作为实现新型城镇化的战略基点，坚持“政策先行、科学规划、产业为基、就业为本、群众自愿”，统筹做好中心镇区和镇域内新型农村社区的规划建设。积极探索完善相关政策，着力解决“地、房、保、钱、管”等5个方面的问题，充分调动基层和群众参与建设的积极性。按照“两个优先”和“五化十有”要求，加快基础设施建设和公益事业发展。坚持多策并举，注重市场运作，创新建设模式，抓好计划启动的77个新型农村社区建设。集中力量，以新型农村社区建设为切入点，加快推进 “三化”协调科学发展试验区建设，为全市作出示范。

加速推进许昌新区建设。按照“四区一基地”的功能定位和“主副两中心、四区加两翼”的空间布局，坚持“四个优先”，全面掀起建设高潮。突出“国际视野、许昌特色”，认真落实新区总体规划、专项规划和主副中心城市设计，高标准、高水平抓好建设。实施“1516”项目投资计划，抓好总投资425亿元的153个重点项目建设。全面加快主、副中心建设，着力推进“十路一轴一湖一廊”等基础设施建设，不断提升承载能力。积极推进社区改造和管理，扎实做好群众就业、就医、入学和社会保障工作，切实维护群众利益。

加快“一中心、五组团、30个中心镇”建设。把中心城市作为以城促产、以城带乡的主导力量，科学谋划借助郑州、强化支撑、加快崛起的重要举措，积极与郑州高效联系、融合发展。加快老城区改造提升、东城区完善提质、开发区提速增效，实施好124个城建重点项目和51个城中村改造项目，分别完成年度投资54.9亿元和85亿元，着力增强中心市区辐射带动能力。合理确定5个组团城市的功能分工，打造比较优势，完善城市功能，实现各展所长、功能互补、错位发展、内涵提升。坚持用现代城市的理念和标准规划建设小城镇，全面启动30个中心镇建设，进一步完善布局、提升水平。加快推进新元大道、许禹快速通道、国道107线长葛北段改建工程，做好许昌至新郑机场、禹州至襄城、长葛至禹州等快速通道建设前期工作，完善中心城市和县（市）组团间的城际快速交通体系。坚持以创建促管理，全力争创全国文明城市，认真落实创建工作3年工作方案，确保一年巩固基础、两年抓好提升、三年全面达标。深入开展城市管理专项整治，积极拓展数字城管应用领域，实现环城公路以内区域和许昌新区全覆盖，全面提升城市数字化、精细化、灯饰化管理水平。

（二）强化主导作用，加快推进产业结构调整

坚持以新型工业化为主导，推动产业转型升级，加快转变经济发展方式，着力提高经济运行质量。

加快产业集聚区发展。围绕“调规划、扩空间、破瓶颈、促跨越、育集群、争位次”，大力实施产业集聚区“2623”提速计划，确保全市产业集聚区建设继续保持全省领先位次。以抓好10亿元以上的重大项目、培育超百亿元的企业（集团）、打造千亿级的产业集群为目标，完善产业集群发展培育导向机制，引导要素向主导产业集中配置，强化上下游企业合作，拉长产业链条，形成规模优势，建设真正意义的产业集聚区。按照“四集一转”要求，推动产业集聚区规划布局调整，扩展发展空间。加快基础性设施、生产性和生活性服务设施建设，提升承载能力、完善服务功能。加快投融资机制创新，认真落实产业集聚区市、县（市、区）两级联席会议制度，坚持月通报、季考核、年奖惩的绩效考评机制，增强制度保障。

着力优化产业产品结构。树立“集约集群集聚、高端高质高效”发展理念，推进信息化与工业化融合发展，深入实施三大主导、四大特色、五大战略性新兴产业发展战略，做强做优主导特色产业，大力培育战略性新兴产业，着力打造“整壮”产业，力争主导产业主营业务收入突破2000亿元，特色产业主营业务收入突破800亿元，战略性新兴产业主营业务收入突破260亿元。以重点领域的优势企业为核心，突出抓好省重大科技专项和企业研发平台建设，加大国家高新技术企业培育和申报力度，深入开展国家知识产权试点城市建设工作，不断提升自主创新能力。大力实施质量提升、品牌带动、标准化战略工程，积极开展知名品牌产业集聚区创建工作，新培育省级名优产品6个以上。加快企业上市步伐，动态调整上市后备企业，力争1至2家企业成功上市。扎实推进重点企业培育工程，坚持完善企业服务长效机制，大力发展实体经济，推动企业做大做强做优，主营业务收入超百亿元的企业达到5家。

大力发展现代服务业。认真实施2012年服务业提升计划，力争全市服务业增加值增长11%。深入推进“3615”现代物流业提速工程，加快6大物流园区和10大特色产业物流中心建设。大力实施“1238文化振兴计划”，着力打造三张文化旅游品牌，重点培育钧瓷、发制品、生态文化和传媒创意四大产业集群，促进文化旅游产业集群发展。加快旅游基础设施建设，推进灞陵桥景区、神垕古镇综合开发等12个重大文化旅游产业项目，完成年度投资12.5亿元。办好各种节会活动，力争全年接待旅游人数和旅游总收入分别增长12%。积极引进外埠股份制商业银行，推进许昌银行增资扩股，加快农村商业银行和新型农村金融机构组建步伐，防范金融风险，做大做强金融产业。规划建设市区商务中心区和县（市）城区特色商务区，打造城区经济发展载体和新的经济增长点。完善主城区、新区服务业专项规划，加快万村千乡市场工程建设，推进“农超对接”，提升商贸服务业发展水平。落实国家房地产相关政策，加快普通商品住房建设，积极促进房地产业健康发展。大力发展信息服务、创意设计、社区服务等新型业态。

扎实推进节能减排。严格落实节能减排目标责任制、一票否决制，完成省定目标任务。抓好重点行业、重点地区节能降耗，实施重点节能工程。加强产业准入管理，加快淘汰落后产能。强化环境影响评价，严格执行水资源管理制度，市级统筹排污总量指标，坚持有扶有控，促进环境容量高效利用。加强饮用水水源地保护，保障人民群众饮水安全。控制污染排放，推进城镇污水处理、垃圾处理、规模化畜禽养殖污染治理等项目建设，充分发挥减排效益。扎实开展清潩河流域环境综合整治，积极推进农村环境连片综合整治。倡导低碳生活，实施低碳经济发展规划，开展公共领域节能和新能源汽车示范推广试点工作。发展循环经济，有序开展碳排放权、排污权有偿使用交易，推动大周镇国家“城市矿产”示范基地建设。

（三）强化基础作用，积极推进新型农业现代化

以建设现代化高标准粮田、培育农业产业化集群、发展农业公司和推进主食产业化为抓手，强科技保发展、强生产保供给、强民生保稳定，夯实新型农业现代化基础。

稳定提升粮食生产。稳定粮食种植面积，提高粮食综合生产能力，全市粮食种植面积稳定在650万亩，粮食产量稳中求增。深入开展高产创建，大力实施高标准粮田“百千万”建设工程，按照“合理规划、综合配套、稳定面积、主攻单产”的原则，建设百亩方18个、千亩方24个、万亩方23个。加强农业技术指导服务，继续实施“万名科技人员包万村”行动，良种覆盖率达到96%以上，测土配方面积达到88%以上，加强重大病虫害监测预警和应急防治。认真落实各项强农惠农富农政策，充分调动农民种粮积极性。

大力发展农业集群。以农业公司为抓手，突出基地化、标准化、规模化、信息化，打造“全链条、全循环、高质量、高效益”现代农业产业化集群。着力抓好优质粮食生产基地、优质花木和中药材基地、优质蔬菜生产加工基地“三个百万工程”。加快农业科技创新，推进许昌国家级农业科技园区建设。在“三化”协调科学发展试验区规划建设20万亩高产农业科技园、5万亩高效农业科技园和5万亩生态农业科技园。突出抓好许昌鄢陵花木产业集群试点建设，全年新增花木面积6.5万亩。加快建设河南省畜牧产业化（生猪集群）襄城实验区，继续推进“千场百区标准化提升工程”和“三带六区五基地”建设，畜牧业产值突破140亿元。抓好粮食生产加工集群建设，全市粮食加工能力达到110万吨。加强规划，完善设施，启动建设百年浓香烟草集群。加大招商引资力度，加快推进许昌农机物流园和粮食物流园建设。大力发展农业公司和农民专业合作社，重点培育市级以上农业产业化龙头企业，新增农民专业合作社100家。

加快基础设施建设。抓住国家加大水利建设的政策机遇，加快农田水利、河道治理、病险水库水闸除险加固等水利基础设施建设，完善乡（镇）、村水利服务体系，提高水资源保障和防洪除涝抗旱能力。突出抓好襄城县北汝河综合治理工程、鄢陵县引黄调蓄工程等12个水利项目，完成农田水利“最后一公里”工程建设34万亩。加强农村水电路气等设施建设，新解决农村居民安全饮水16.3万人，改造农村公路300公里，新建农村户用沼气1万口、大中型沼气工程5座，完成新一轮农村电网提升改造工程，推进农村公益事业建设“一事一议”财政奖补工作，改善农村生产生活条件。扎实做好南水北调干线征迁、配套工程建设和移民后期扶持工作。持续提升林业生态建设水平，认真筹办第十二届中原花木交易博览会，完成林业生态建设22万亩。

（四）强化项目带动，增强经济发展支撑

按照提升“三个比重”、做到“三个一批”的要求，加快重点项目建设，保持投资稳定增长，持续增强经济增长拉动力。

大力实施“1241”投资促进计划。切实抓好120个重点项目，完成年度投资超过400亿元，带动全市固定资产投资超过1000亿元。其中工业项目80个，年度计划投资210亿元；现代服务业项目18个，年度计划投资32亿元；社会事业项目5个，年度计划投资7.7亿元；交通电力项目8个，年度计划投资29亿元；农林水利项目5个，年度计划投资17亿元；城建打捆项目4个，年度计划投资114亿元。通过实施“1241”投资促进计划，使重点项目投资占固定资产投资的比重提高到40%以上，工业项目投资占重点项目投资的比重提高到52%以上，服务业项目投资占重点项目投资的比重提高到10%以上。

着力推进前期项目。建立完善重大前期谋划项目储备推进机制，依托园区、现有产业、大型企业，推进重大项目谋划实施，优质重大前期项目储备规模达到50个。扎实做好项目前期工作，强化运作，交叉推进，突破难点，力促前期项目早日落地开工。

切实加强项目运作。服务方面，坚持联审联批、“一站式”服务，高效、快捷地办理各类手续；融资方面，突出市场运作，强化招商引资，积极争取信贷支持，推动企业上市，激活民间资本，抓好政府投融资平台建设，多渠道筹措资金；资源配置方面，对新上项目和企业在投资强度上设置门槛，实现土地等资源集约节约利用、集群集约发展。进一步研究完善重大项目考核、激励办法，着力解决重大项目偏少问题。加强与国家、省有关部门沟通对接，力争更多重点项目进入国家、省计划盘子。

完善落实推进机制。坚持完善市级领导联系重点项目制度、重点项目联动和资源配置机制、重点项目联审联批制度等10项行之有效的推进机制，及时协调解决项目建设问题。建立完善重点项目动态管理机制，根据客观情况变化，适时调整重点项目计划盘子。不断优化项目建设环境，严厉打击阻工、扰工、强买强卖等不法行为。

（五）强化改革开放，增添发展活力动力

把改革开放作为应对复杂局面和破解瓶颈制约最直接、最有效、最综合的全局性举措，继续深化改革开放，努力实现新的突破。

提升对外开放水平。把招商引资、承接产业转移作为开放工作的重中之重，突出“招大引优”，大力实施“5516”招商引资行动计划，围绕园区、产业、企业、项目和重点区域等5个方面，谋划推介500个重点招商项目，合同引进市外资金1000亿元，重点抓好市级领导联系的60个投资亿元以上的招商项目。扎实开展“招商引资项目落地年”活动，强化签约项目跟踪落实，确保早建设、早投产、早见效。拓展开放的广度和深度，吸引境内外投资者进入农业、现代服务业、基础设施、城乡建设和文化旅游等领域。优化开放环境，提升服务水平，重诺守信，营造亲商、安商良好氛围。抓好出口基地建设，大力引进出口型项目，加快转变外贸发展方式，巩固发制品出口优势，加大机电和高新技术产品等出口份额，促进出口稳定增长、结构优化升级。积极推进海关、许昌新区出口加工区申报工作，着力构建大开放格局。

深化重点领域改革。扎实推进30家企事业单位改革改制，着力解决历史遗留问题，积极引进战略合作者，推动产权改革、转企改制和战略重组。按照“政府主导、市场运作、为民惠民、公益优先”的原则，突出抓好城市公交公司化改造。扎实推进医疗卫生体制改革，完善基本药物制度，促进公共卫生服务均等化。持续推进行政审批制度改革，巩固提升招投标体制改革成果。稳妥推进事业单位分类改革，有计划、有步骤地完成清理规范、科学分类。深化文化体制改革，推动文艺院团等文化团体整合重组。继续深化农村改革，推进农村土地适度规模经营，土地流转面积达到135万亩。

破解发展瓶颈制约。创新建设用地保障机制，落实城乡建设用地增减挂钩各项政策措施，严格清查批而未用土地，完善土地节约集约利用标准化体系和考核评价体系，促进土地高效利用，着力解决建设用地刚性需求与耕地保护硬性约束的矛盾；创新资金保障机制，完善提升政银企洽谈对接机制，加大资本和货币市场融资工作力度，扩大信贷投放，集聚民间投资效应，破解小微企业融资难题，力争全年融资210亿元；创新拆迁推进机制，积极探索拆迁补偿安置模式、拆迁监管办法和拆迁改造方式，合理设置补偿标准，维护合法利益，努力实现群众、企业和社会等多方共赢；创新煤电运联调联动机制，大力发展热电联产，推进坚强智能电网建设，突出抓好许昌新区、产业集聚区电网建设和老城区电网改造，切实保障电力供应。

（六）强化民生民本，促进社会和谐进步

适应人民群众新期盼，加大财政民生投入，实施“十项民生工程”，让人民群众得到更多实惠。

千方百计扩大就业。坚持以创业带动就业，落实优惠政策，扩大小额担保贷款发放规模，争创国家级创业型城市。深入推进全民技能提升工程，全年培训20万人。按照“扩增岗位、提升技能、健全机制、完善服务”的要求，实施更加积极的就业政策，新增农村劳动力转移就业7万人，下岗失业人员再就业1.5万人，就业困难人员再就业8500人，保持城镇零就业家庭动态为零。强化就业公共服务，重点抓好高校毕业生、城镇就业困难群体、退役人员和残疾人就业帮扶工作。构建和谐劳动关系，维护劳动者合法权益，落实最低工资标准和企业工资集体协商制度，稳步提高城乡居民收入。

大力推进社会建设。优先发展教育事业，深入实施学前教育3年行动计划，完成40所公办幼儿园建设任务；认真落实“两免一补”政策，启动“1559”教育信息化工程，抓好138个校安工程项目，新建扩建8所市区小学，保障农民工子女就近入学，推进义务教育学校办学条件标准化建设；深入推进职教攻坚，加快电气职业学院二期工程建设，建成5个县级职教中心。着力提高医疗卫生保障水平，实施医疗机构倍增计划，促进新增医疗卫生资源向农村倾斜，巩固提升标准化村卫生室建设成果，继续抓好重大传染病防控和救治工作。促进文化事业繁荣发展，扎实推进文化惠民工程，加快市广电大厦、县级文化馆等基础设施建设，做好许昌塔文化博物馆和禹州中医药文化博物馆筹建工作，实现各行政村农家书屋全覆盖。扎实推进体育器材进社区和农民健身工程，提高人民群众身体素质。积极做好市科技馆、鹿鸣湖体育中心各项前期工作，争取早日开工。落实计划生育利益导向机制，稳定低生育水平，促进人口均衡发展。

完善社会保障体系。按照“广覆盖、保基本、多层次、可持续”的方针，加快建立覆盖城乡居民的社会保障体系。加大社会保险扩面征缴力度，力争各项社会保险基金应收尽收。大力发展社会福利和慈善事业、妇女儿童事业、老龄事业、残疾人事业。落实“分类指导、科学规划、优化结构、讲求实效、强力推进”的要求，加快保障性住房建设，完成省定14397套各类保障性住房建设任务，制定完善分配、管理和退出等制度，确保工程质量和分配公平。健全社会救助标准与物价上涨联动机制，城市低保标准提高到每人每月260元，农村低保标准提高到每人每年1500元。落实国家10年扶贫开发纲要，完成20个贫困村整村推进建设任务。提升农村敬老院管理水平，力争20所达到省级文明敬老院标准。

推进社会管理创新。健全“党委领导、政府负责、社会协同、公众参与”的社会管理格局，强化基层社会服务管理功能，提升社会管理科学化水平。建立覆盖全社会的征信系统，强化各种中介组织监管，推进社会信用体系建设。加快无线城市建设，推动信息技术在政务、商务、生产生活、社会管理等领域的应用。巩固煤炭企业兼并重组成果，确保煤矿安全生产。大力开展“安全许昌”创建活动，加强交通、校园、校车、消防、食品、非煤矿山、特种设备、危险化学品等重点领域的安全监管。深入实施“六五”普法依法治理规划，加强法制宣传和法律援助。深化“平安许昌”创建，不断改进信访工作，夯实基层基础；继续加强社会治安综合治理，依法打击各种违法犯罪活动，维护社会大局和谐稳定。

加强国防和民兵预备役工作，促进军民融合式发展。重视和支持工会、共青团、妇联等人民团体工作。切实做好外事、侨务、对台工作。继续做好地方史志、新闻出版、机关事务、民族、宗教、人事、审计、统计、档案、地震、气象、人防等各项工作。

四、全面加强政府自身建设

圆满完成今年乃至今后五年的任务，必须加快领导方式转变，认真落实“三具两基一抓手”，深入推进“两转两提”，切实增强推动科学发展能力，着力建设人民满意的政府。

（一）着力提升依法行政水平。严格依照法定权限和程序行使权力、履行职责，维护宪法和法律的权威。依法接受人大及其常委会的法律监督、工作监督，主动接受政协的民主监督，认真办理人大代表建议和政协委员提案。坚持依法民主决策，提高科学决策水平。完善行政执法程序，规范行政执法行为。加强行政复议工作，强化行政执法责任和过错追究。健全行政监督体系，严格行政问责。

（二）着力提升科学运作水平。运作是重要的执政能力，是干的能力、做的能力、创造性抓落实的能力。面对新形势、新挑战，必须遵循规律重运作，按照客观规律办事，加强各项工作实施过程的程序化、规范化管理，做到规范操作、快捷高效；必须创新方法善运作，与时俱进，解放思想，转变观念，不拘泥于条条框框限制，不墨守老经验、老办法，不断在思想方法、领导方法、工作方法上有所创新；必须把握契机快运作，找准解决问题的切入点，不失时机地破解关键性、疑难性问题，推动工作开展，实现既定目标。

（三）着力提升务实发展水平。坚持群众路线，深入实际，依靠群众，求真务实，力戒形式主义、官僚主义，说到做到、说好做好。完善跟踪落实机制，对重大事项和重点工作，雷厉风行、锲而不舍、一抓到底。强化责任，勇于担当，做到在其位、谋其政、司其职、尽其责、有作为。精简会议文件，改进会风文风，减少迎来送往，腾出更多精力用于发展经济、改善民生。

（四）着力提升执政为民水平。牢固树立宗旨观念，深入开展创先争优活动，争做“人民满意的公务员”，努力建设一支政治坚定、业务精湛、作风过硬的公务员队伍。加强政府领导班子和领导干部廉政建设，严格落实党风廉政建设责任制，扎实推进惩治和预防腐败体系建设。坚持用制度管人、管权、管事，始终把权力运行置于阳光之下。大力倡导艰苦奋斗、勤俭节约，坚决反对和制止奢侈挥霍、铺张浪费行为。加强监察、审计工作，对重要领域和关键环节实施重点监督，严肃查处各类违法违纪案件，依法严惩腐败分子。

各位代表！过去的辉煌成就令人鼓舞，未来的宏伟蓝图催人奋进。让我们在中共许昌市委的坚强领导下，振奋精神，团结一致，开拓创新，务实发展，圆满完成各项目标任务，致力在中原经济区建设中率先崛起、富民兴许，以优异成绩迎接党的十八大胜利召开
